# Visuospatial Sequence Learning without Seeing

**DOI:** 10.1371/journal.pone.0011906

**Published:** 2010-07-30

**Authors:** Clive R. Rosenthal, Christopher Kennard, David Soto

**Affiliations:** 1 Department of Clinical Neurology, University of Oxford, Oxford, England, United Kingdom; 2 Department of Clinical Neuroscience, Imperial College London, London, England, United Kingdom; Harvard Medical School, United States of America

## Abstract

**Background:**

The ability to detect and integrate associations between unrelated items that are close in space and time is a key feature of human learning and memory. Learning sequential associations between non-adjacent visual stimuli (higher-order visuospatial dependencies) can occur either with or without awareness (explicit vs. implicit learning) of the products of learning. Existing behavioural and neurocognitive studies of explicit and implicit sequence learning, however, are based on conscious access to the sequence of target locations and, typically, on conditions where the locations for orienting, or motor, responses coincide with the locations of the target sequence.

**Methodology/Principal Findings:**

Dichoptic stimuli were presented on a novel sequence learning task using a mirror stereoscope to mask the eye-of-origin of visual input from conscious awareness. We demonstrate that conscious access to the sequence of target locations and responses that coincide with structure of the target sequence are dispensable features when learning higher-order visuospatial associations. Sequence knowledge was expressed in the ability of participants to identify the trained higher-order visuospatial sequence on a recognition test, even though the trained and untrained recognition sequences were identical when viewed at a conscious binocular level, and differed only at the level of the masked sequential associations.

**Conclusions/Significance:**

These results demonstrate that unconscious processing can support perceptual learning of higher-order sequential associations through interocular integration of retinotopic-based codes stemming from monocular eye-of-origin information. Furthermore, unlike other forms of perceptual associative learning, visuospatial attention did not need to be directed to the locations of the target sequence. More generally, the results pose a challenge to neural models of learning to account for a previously unknown capacity of the human visual system to support the detection, learning and recognition of higher-order sequential associations under conditions where observers are unable to see the target sequence or perform responses that coincide with structure of the target sequence.

## Introduction

A key feature of the neurocognitive mechanisms that support abilities such as motor skill learning, declarative memory, and language acquisition is the capacity to detect and integrate associations between previously unrelated items [1], [2], [3]. The learning of non-adjacent sequential stimuli (higher-order dependencies), independently of non-specific perceptual-motor skill learning, has been extensively studied using the serial reaction time task (SRT task) [4], [5], [6]. Learning on the SRT task typically involves sequential manual key presses [7], eye movements [8], and/or covert reorienting of visuospatial attention [9], [10] directed to four fixed locations in response to visual targets that appear at one of four corresponding spatial locations. Visuospatial sequential associations can also be learned via simple observation of a target sequence [11], [12], [13]; however, learning under these conditions is not confined to the coding of associations between the visual stimuli (pure perceptual-based learning) because motor responses in the form of eye movements to the target sequence were not precluded during training [14]. Similarly, in studies that have reported learning under conditions where the dimension for manual responses is uncorrelated with the sequence of target locations (e.g., target identity, as opposed target location) [15], [16], [17], [18], [19], it not possible to exclude a functional role for eye movements in the orienting responses to the target locations. Furthermore, all prior demonstrations of sequence learning, either with or without awareness of the products of learning (explicit vs. implicit learning), have been dependent on observers having conscious access to the sequence of target locations during learning. Therefore, even if learning occurs under an incidental orientation to the target sequence and the products are unavailable to conscious awareness (i.e., implicit learning) [20], it is not currently possible to determine the extent to which higher-order sequential associations can be learned independently of awareness for the locations of the target sequence.

**Figure 1:**
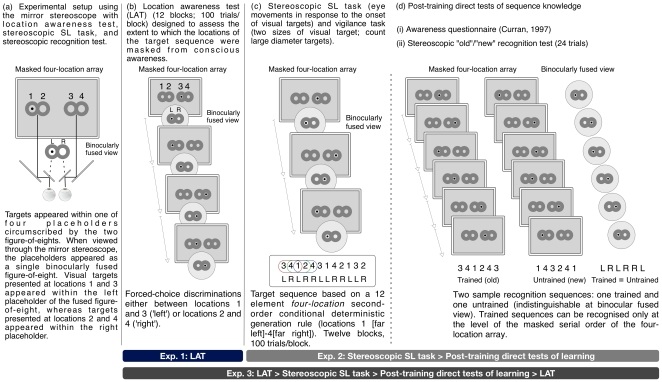
Experimental setup and design. (a) Visual stimuli were viewed through a mirror stereoscope that was calibrated to ensure that the four-target locations of the target sequence appeared as two locations in the binocularly fused view; (b) The location awareness test (LAT) administered in experiment 1 assessed whether or not observers could use information about the mapping of targets to perform accurate forced-choice discriminations between masked locations 1 and 3 and locations 2 and 4, when targets were viewed through a mirror stereoscope. In experiment 3, a shortened LAT was administered prior to training on stereoscopic SL task and after the recognition test. (c) Learning on the stereoscopic SL task was examined under conditions of incidental (exp. 2) and intentional (exp. 3) orientation to the mapping between the masked four-target locations and two locations of the binocular fused view. The target sequence followed a deterministic second-order conditional rule presented at the level of the four masked locations (the same sequence structure was also used in experiment 1). Sustained attention to targets was ensured by instructing participants to maintain a block-wise cumulative count of large diameter targets (presented randomly in place of standard diameter targets; the LDT counting task). Responses on the stereoscopic SL task were confined to eye movements (and/or covert reorienting of visuospatial attention) to the two target locations in the binocular fused view; and (d) Post-training direct tests of learning (exps. 2 and 3): (i) sequence awareness questionnaire; and, (ii) stereoscopic recognition test showing one trained (“old”) trial and one (“new”) untrained trial, each comprised of six-item sequence - responses during the presentation of each six-item sequence were confined to simple observation. Trained and untrained sequences differed only at the level of the masked four-location array. Participants were asked to determine whether each short-sequence was “old” or “new” and then rate their confidence on a 6-point scale.

Here, we report the results from a novel (stereoscopic) sequence learning (SL) task and a novel stereoscopic recognition test that we developed to investigate pure perceptual-based learning under conditions where there was no awareness of the target sequence and no correlation between the structure of the stimulus sequence and structure of the orienting (oculomotor and/or covert reorienting of visuospatial attention) responses to the visible targets—training did not involve manual responses. Each target on the stereoscopic SL task appeared at the centre of one of four placeholders circumscribed by two horizontally aligned figure-of-eights (read from left to right, placeholders 1 and 2 were in the left figure-of-eight and placeholders 3 and 4 were in the right figure-of-eight; targets appeared for 1000 ms; see Figure 1a). When viewed through a mirror stereoscope, the placeholders appeared as a single binocularly fused figure-of-eight aligned along the horizontal meridian. Visual targets presented at locations 1 and 3 appeared within the left placeholder of the fused figure-of-eight (‘left’ targets), whereas targets presented at locations 2 and 4 appeared within the right placeholder (‘right’ targets). Hence, on any single trial, the placeholders presented to each eye were identical, whereas the visual target appeared as a dichoptic stimulus (i.e., was presented to the separate and independent field of view of one eye), with the eye-of-origin of visual input masked from conscious awareness [21], [22], [23], [24] (Figure 1a).

The order of the target sequence was based on a deterministic second-order conditional generation rule involving the four-locations, where, at the lowest structural level, the ability to predict a target location was dependent on learning two preceding target locations [25] (see Figure 1c and the Methods section for further details). Hence perceptual learning involved the development of sensitivity to a sequence of associations between non-adjacent—higher-order—stimuli, as opposed to the simpler pairwise sequential associations between adjacent stimuli that characterises a first-order sequence. Importantly, however, these higher-order sequential associations could not be consciously perceived because the sequence was based on the four locations that were masked by binocular fusion of the stimuli and responses were directed to two locations. Other salient features of the target sequence such as the simple frequency of positions, first-order transition frequency, and reversal frequency (e.g., 1-2-1) were controlled to orient learning towards the second-order conditional rule [25]. An important feature of learning such higher-order (vs. simpler pairwise) associations on the SRT task is that it typically engages a distinct cortico-striatal/cortico-cerebellar network, with activation in the inferior parietal lobule (IPL) identified with coding an effector-independent (visual/spatial coordinates) description of successive locations [26], [27], whereas the coding of higher-order visuospatial dependencies, per se, has been identified with activation within the hippocampus and related structures [7], [8], [28].

In experiment 1, we obtained an objective and independent measure of the efficacy of masking the locations of the target sequence. In particular, participants were assessed to determine if they could use information about the mapping between the four-target positions and the two positions of the binocularly fused figure-of-eight to perform accurate forced-choice discriminations between masked locations 1 and 3 and locations 2 and 4 (Figure 1b)—the so-called, location awareness test (LAT). In experiment 2, participants were trained on the masked higher-order visuospatial sequence under conditions where the target stimuli were presented using a mirror stereoscope and the responses were confined to eye movements and/or covert reorienting of visuospatial attention (Figure 1c). Unlike the manual SRT task, where the emergence of sequence-specific knowledge can be assessed using an indirect latency and/or accuracy based measure administered during training, sequence-specific knowledge was assessed by means of an ‘old’(trained)/‘new’(untrained) recognition test administered after training (see Figure 1d). Sensitivity to even partial declarative knowledge related to the target sequence was ensured by using a six-point scale to obtain a confidence rating for each ‘old’/‘new’ recognition decision and by reinstating the conditions of the learning environment on each short old/new recognition probe [10], [29]. Experiment 3 was a replication of experiment 2 with the exception that participants performed a shortened LAT before training on the stereoscopic SL task and after completion of the recognition test. This manipulation enabled us to examine two additional issues: (1) does an intentional orientation to the mapping between the four-target locations and two locations of the binocularly fused figure-of-eight have an effect on the ability to learn the target sequence; and (2) does exposure to the target sequence during training and test modulate the ability of participants to consciously perceive the masked target locations.

## Methods

### Participants

Twenty-eight participants were recruited (M = 27.5 years; 19 females). Eight participants took part in experiment 1, 10 participants took part in experiment 2, and 10 participants took part in experiment 3. All participants had normal or corrected-to-normal visual acuity and received a payment of £15. None of the participants had previous experience of the SRT task or sequence learning tasks.

### Ethics Statement

Local research ethics committee (Hammersmith Research Ethics Committee Reference: 04/Q0406/147) approval was granted for the experimental procedures. All participants provided written informed consent for the collection of data and subsequent analysis.

### Visual Stimuli

Visual stimuli were presented on a 21” Sony Trinitron CRT computer monitor configured to a refresh rate of 100 Hz and to a screen resolution of 1024×768 pixels. Target stimuli on the location awareness test, stereoscopic SL task and stereoscopic recognition test appeared within four circular placeholders (2 cm in diameter; subtending 1.9° of visual angle) circumscribed by two horizontally oriented figures-of-eight (7.3 cm in length [subtending 7.0° of visual angle] ×4.5 cm in height [subtending 4.3° of visual angle]) positioned along the horizontal meridian of the computer monitor on a black background (Figure 1a). The viewing distance was 60 cm. The left and right figures-of-eight were presented independently to the left and right eyes, respectively, by means of a mirror stereoscope (GeoScope™ Pro, Stereoaids, Australia). Visual input to each eye was constrained by the field of view provided by the two eye-pieces of the mirror stereoscope (see Figure 1a).

### Design

#### Experiment 1: Location awareness test

A location awareness test (LAT) was administered to assess whether or not observers could use information about the mapping of targets to perform accurate forced-choice discriminations between masked positions 1 and 3 and positions 2 and 4, when targets were viewed through the mirror stereoscope. Therefore, the LAT was used as an assay of the efficacy with which the four spatial locations used to present the target sequence were masked by the stereoscopic presentation. The LAT was comprised of 12 blocks of trials (100 trials/block), each involving trial-wise forced-choice discrimination between locations 1 and 3 for targets that appeared within the left placeholder and between locations 2 and 4 for targets that appeared the right placeholder of the binocularly fused figure-of-eight (Figure 1b).

#### Experiments 2 and 3: Stereoscopic SL task, large diameter target counting (vigilance) task, and stereoscopic recognition test

Experiment 2 was comprised of two phases (see Figure 1c and 1d): (1) a training phase consisting of a stereoscopic SL task performed alongside a concurrent vigilance task (the large diameter target counting task); and (2) a post-training direct test phase comprised of a sequence awareness questionnaire [30] and a stereoscopic recognition test. Experiment 3 was a conceptual replication of experiment 2 with exception that participants performed a LAT before the training phase and after completion of the recognition test. The pre-training and post-test LATs comprised three-blocks of 24 trials each. The targets presented on the pre-training and post-test LATs followed a pseudorandom sequence such that equal frequencies of occurrence were used for each of the four locations and there were no contiguous repetitions of a single location.

Training on the stereoscopic SL task involved the presentation of 12 blocks of trials after an initial short sequence designed to ensure stable fusion of the stimulus array (Figure 1c). Each block of the stereoscopic SL task was comprised of 100 trials; the first four trials were buffers and were followed by eight repetitions of one of the two 12-element second-order condition (SOC) sequences of target locations (SOC1: 3 4 2 3 1 2 1 4 3 2 4 1; SOC2: 3 4 1 2 4 3 1 4 2 1 3 2; positions 1–4 are read from left to right of the masked four-location array, with the spatial locations corresponding to numeric values of the SOC sequence).

The two sequences were identical to those used by Destrebecqz and Cleeremans [31] and were generated in accordance with a deterministic second-order conditional generation rule, where, at the lowest structural level, the ability to predict a target location is dependent on learning two preceding target locations [25]. Both of these sequences were equated along salient sequential constraints of simple frequency, first-order transition frequency, reversal frequency (e.g., 1-2-1), and rate of full coverage. Therefore, the sequences differed only at the level of three or more consecutive locations, and, unlike a first-order sequence, performance cannot improve from learning the frequencies of individual locations or pairs of locations. Half of the participants were trained on SOC1 and the other half were trained on SOC2; SOC1 and SOC2 were also counterbalanced for use as the stimulus materials in the 12 blocks administered in experiment 1. Critically, when viewed through the mirror stereoscope, the sequences occupied the following sequence of left(L)/right(R) locations in the binocular fused view: SOC1: L R R L L R L R L R R L; and SOC2: L R L R R L L R R L L R (Figure 1c).

A large diameter target counting (vigilance) task (LDT counting task) was performed concurrently with stereoscopic SL task [10]. Visual targets during training consisted of black circles of two sizes—a standard (5 mm diameter) and a large diameter target (LDT; 8 mm diameter)—that appeared within one of the four placeholders locations. The LDT counting task was designed to ensure that participants sustained their attention to the stimuli. LDTs were presented between 18% and 36% on each block of the stereoscopic SL task—the order of the LDTs was random within a block and set at a proportion that ensured the participants were able to perform at ceiling.

Post-training direct tests of sequence knowledge (sequence awareness questionnaire, stereoscopic recognition test) immediately followed the training phase. The sequence awareness questionnaire involve the selection of one of five propositions: 1 = “The sequence of stimuli was random”; 2 = “Some positions occurred more often than others; 3 = “The movement was often predictable”; 4 = “The same sequence of movement would often appear”; and 5 = “The same sequence of movements occurred throughout the experiment” [30].

The stereoscopic recognition test followed the questionnaire and was comprised of 12 trained (old) and 12 untrained (new) six-item sequences (Figure 1d). Twelve sequences (starting from each ordinal position of the 12-element SOC sequence for six consecutive locations) were generated from SOC1 and 12 were generated from SOC2. Therefore, the second-order conditional serial order of the trained and untrained sequences were different, but fundamentally, at the conscious level of the binocular fused view, the trained and untrained sequences were matched across all dimensions and their appearance was identical (see Table 1 and Figure 1d). A six-point scale was used to obtain a confidence rating for each six-item sequence so that participants could express even partial declarative knowledge [29]: 1 = “I'm certain that this fragment was part of the training sequence”; 2 = “I'm fairly certain that this fragment was part of the training sequence”; 3 = “I believe that this fragment was part of the training sequence”; 4 = “I believe that this fragment was not part of the training sequence”; 5 = “I'm fairly certain that this fragment was not part of the training sequence”; and 6 = “I'm certain that this fragment was not part of the training sequence.”

**Table 1:** Masked positions of target stimuli based on the two second-order conditional sequences – SOC1 and SOC2 – presented on the recognition test.

Stereoscopic Recognition Test		
Masked Locations (SOC1)	Masked Locations (SOC2)	Target Locations: Binocular Fused View
3 4 2 3 1 2	1 4 2 1 3 2	L R R L L R
4 2 3 1 2 1	4 2 1 3 2 3	R R L L R L
2 3 1 2 1 4	2 1 3 2 3 4	R L L R L R
3 1 2 1 4 3	1 3 2 3 4 1	L L R L R L
1 2 1 4 3 2	3 2 3 4 1 2	L R L R L R
2 1 4 3 2 4	2 3 4 1 2 4	R L R L R R
1 4 3 2 4 1	3 4 1 2 4 3	L R L R R L
4 3 2 4 1 3	4 1 2 4 3 1	R L R R L L
3 2 4 1 3 4	1 4 2 1 3 2	L R R L L R
2 4 1 3 4 2	2 4 3 1 4 2	R R L L R R
4 1 3 4 2 3	4 3 1 4 2 1	R L L R R L
1 3 4 2 3 1	3 1 4 2 1 3	L L R R L L

Trained/untrained status of each set of 12 six-item sequences was determined by training on the stereoscopic SL task (SOC1 or SOC2). Binocular positions for SOC1 and SOC2 are identical across matched pairs of the six-item recognition sequences. Masked locations 1, 2, 3, 4, read from left to right for masked four-location placeholder array. L  =  Left placeholder; R  =  Right placeholder of the binocular fused view.

All of the behavioural tasks were implemented and administered using E-prime (v2.0, Psychology Software Tools, Inc., PA, USA).

### Procedure

Participants were tested individually and all three experiments were performed in a dark visual Ganzfeld. A chin rest was used to maintain head position throughout the experiment. Initial calibration involved moving the two figure-of-eights along the horizontal meridian to determine the separation necessary to achieve a stable, fused figure-of-eight. Participants were presented with a short sequence of targets (5 mm diameter black circles) to establish whether or not the location of the targets was reliably and accurately mapped between the four-location placeholder array and two placeholders of the binocularly fused figure-of eight. In particular, we assessed whether visual targets presented at positions 1 and 3 appeared within the left placeholder of the fused figure-of-eight, and whether targets presented at positions 2 and 4 appeared within the right placeholder. Calibration was performed before each block of trials on the location awareness test and stereoscopic SL task and immediately prior to the stereoscopic recognition test.

#### Location awareness test (LAT) (Experiments 1 and 3, Figure 1b)

Participants were instructed on the mapping between the location of each visual target within the two figures-of-eight and the two locations within the binocularly fused fight-of-eight. In particular, participants were informed that the left figure-of-eight circumscribed placeholder locations 1 and 2 and projected to the left eye, whereas the right figure-of-eight circumscribed locations 3 and 4 and projected to the right eye. It was also explained that both figures-of-eight were binocularly fused due to stereoscopic presentation and that each trial of the LAT test would involve the presentation of a single target that could appear at positions 1, 2, 3 or 4, but that targets presented at positions 1 and 3 would appear within the left placeholder whereas targets presented at positions 2 and 4 would appear within the right placeholder.

Participants were instructed to respond with a key press on a four-button response pad that mapped to the four-position array of visual targets (Serial Response Box, Psychology Software Tools, Inc, Pittsburgh, USA). Specifically, participants were required to identify the location of each target and indicate their response by pressing either button 1 or 3 if a target appeared on the left of the fused figure-of-eight or respond with button 2 or 4 if a target appeared on the right. Responses to locations 1 and 2 were made with the middle and index fingers of the left hand, respectively, and to locations 3 and 4 with the index and middle fingers of the right hand, respectively. On detection of a response, the target stimulus was extinguished and the next target was presented.

#### Training phase: Stereoscopic SL task and concurrent LDT counting (vigilance) task (Experiments 2 and 3, Figure 1c)

Each trial of the stereoscopic SL task involved the presentation of a target stimulus at the centre of one of the four enclosed regions circumscribed by the two horizontal figure-of-eights (1000 ms). Extinction of each target was followed by a 200 ms interval. Participants were instructed to attend to the location of each target, maintain a cumulative count of LDTs, and report the value at the end of each block of training on the stereoscopic SL task. On-screen feedback was provided at the end of each block of trials and was based on the actual number of LDTs: participants responding with a value within 5% accuracy were informed that their count was accurate and were asked to continue with their good performance, whereas participants responding with a count of 5% error or greater were shown their percentage of under- or over-estimation and were instructed to try harder on the forthcoming block of trials.

#### Post-training direct test phase: sequence awareness questionnaire and stereoscopic recognition test (Experiments 2 and 3, Figure 1d)

After selecting a response on the sequence awareness questionnaire to indicate the extent to which regularities had been detected during the training session, participants were informed that the target stimuli had followed a regular repeating sequence during the training session. Participants were instructed to attend to the presentation of each six-item sequence on the stereoscopic recognition test in the same way as on the stereoscopic SL task (Figure 1d) and then respond using a key press to indicate whether the sequence was ‘old/seen’ or ‘new/unseen’; that is, participants were asked to decide whether or not each short-sequence had appeared during the training session. After each ‘old’/‘new’ discrimination, participants were asked to rate how confident they were in their judgement on the 6-point scale [29].

### Data analysis of the LAT

The proportions of correct discriminations (hits) and false alarms on the LATs were computed as follows. For each perceptual discrimination, one of the responses (e.g., ‘1’) was treated as ‘signal present’ and the other response (e.g., ‘3’) as ‘signal absent’. Thus, responding with a key press at position ‘1’ to targets at position 1 were labelled as correct responses or ‘hits’, whereas responding with a key press at position ‘1’ to targets at position ‘3’ were recorded as ‘false alarms’. The same procedure was applied in the case of ‘right’ targets at positions 2 and 4. In this way, we obtained the probability of hits – P(H) – and false alarms – P(FA) - to calculate a measure of perceptual sensitivity, d', based on signal detection theory [32].

## Results

### Experiment 1: Efficacy of masking location information for the higher-order visuospatial sequence

**Figure 2:**
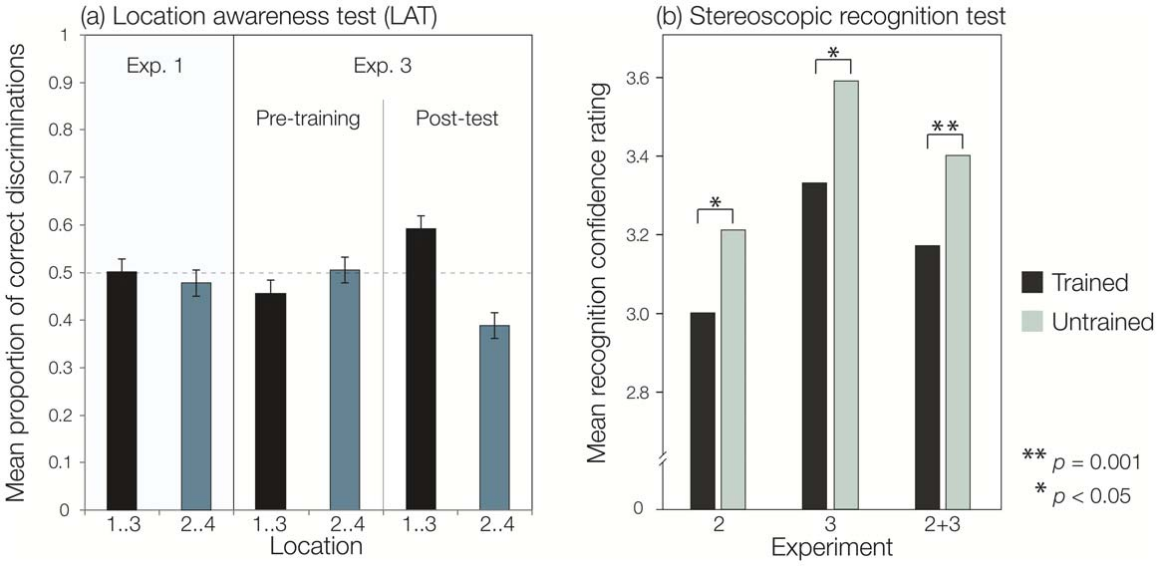
Performance on the location awareness test (LAT) and post-training recognition test. (a) Mean of proportion correct discriminations on the LAT (exps. 1 and 3), with S.E.M. In experiment 1, the results from the LAT revealed that participants were unable to identify target locations above chance (hashed line). In experiment 3, the results revealed that sensitivity to the location of visual targets remained at chance even after training on the stereoscopic SL task and administration of the direct tests of sequence knowledge. (b) Mean recognition confidence ratings assigned to the 12 trained and 12 untrained six-item sequences are shown for experiments 2 (incidental learning and orientation to the masked four-position complex sequence) and 3 (incidental learning but intentional orientation to mapping between the four-location array and binocular fused view). Trained sequences were allocated a rating between 1 and 3, whereas untrained sequences were allocated a rating between 4 and 6. Results from experiments 2 and 3 are combined because there was no interaction between performance on the recognition test and orientation to the mapping between the binocular fused view and the four-location array, *F*_(1,18)_ <1.

The mean proportions of correct responses on the LAT were consistent with a failure to discriminate between targets that appeared at locations 1 and 3 (M = 0.50, S.E.M. = 0.04) and at locations 2 and 4 (M = 0.48, S.E.M. = 0.03), and did not differ from chance (0.5) (*t*_(7)_  = 0.68, p = 0.95, and, *t*_(7)_ = 0.70, p = 0.51, for ‘left’ and ‘right’ targets, respectively; see Figure 2a). Similar results were obtained when using a measure of perceptual sensitivity (d') based on signal detection theory [32]. Performance indexed by d' did not differ from chance (d' = 0; Table 2). Participants were, therefore, unable to use knowledge about mapping between the four-target locations and two locations of the binocularly fused figure-of-eight and eye-of-origin information to identify the location of the masked targets (eye-of-origin for positions 1 and 2 was the left eye, whereas the eye-of-origin for positions 3 and 4 was the right eye). These results are consistent with the view that eye-of-origin information involves monocular cells within primary visual cortex that typically exhibit a poor correlation with measures of conscious awareness [33]. Results from experiment 1 thus demonstrate that stereoscopic presentation masked the location of targets within the four-location array, under conditions where salient parameters were matched to those used in experiments 2 and 3. These include the rate of presentation, the number of trials, and the structure of the underlying sequence.

**Table 2:** Mean proportion of hits [P(H)], proportion of false alarms [P(FA)], and d' scores in the LAT administered in Experiment 1.

	P(H)	P(FA)	d'	*t_(7)_*	*p*
‘Left’ targets	0.55	0.55	0.04	0.18	0.86
‘Right’ targets	0.41	0.44	−0.11	−0.69	0.51

Performance was assessed by calculating d', an index of perceptual sensitivity based on signal detection theory. For each perceptual discrimination, one of the responses (e.g., ‘1') was treated as ‘signal present’ and the other response (e.g., ‘3’) as ‘signal absent’. Thus, responding with ‘1’ to targets at position 1 were labelled as ‘hits’ whereas responding with ‘1’ to targets at position ‘3’ were recorded as false alarms. The same procedure was applied in the case of ‘right’ targets at positions 2 and 4. In this way, we obtained the probability of hits – P(H) – and false alarms – P(FA) – to calculate d'. One-sample t-tests indicated that sensitivity scores did not differ from chance (d' = 0).

#### Experiments 2 and 3: Performance on the LDT counting (vigilance) task, evidence of sequence-specific knowledge on the stereoscopic recognition test, and the effect of an intentional orientation to the masked stimulus locations on recognition test performance LDT counting task performance

Two sizes of visual target - a standard target and a large diameter target (LDT) - were presented as part of the novel stereoscopic SL task to ensure that participants maintained attention to the stimuli [34] (see Figure 1c and Methods). Performance on the LDT counting task in experiments 2 (M error across 12 blocks of trials  = 2.04%, S.E.M = 1.01) and 3 (M error across 12 blocks of trials  = 0.82%, S.E.M = 0.21) indicates that participants were able to sustain attention to the stimulus sequence and reliably discriminate between LDTs and standard targets. Importantly, the effortful processing associated with the LDT counting task, by definition, was not directed at learning spatially-contingent responses related to the target sequence. Furthermore, performance on the LDT counting task was consistent with a level of automaticity that would have allowed resources to be directed at learning [35]. Indeed, behavioural evidence indicates that secondary tasks such as tone counting disrupt performance, but not learning, on the manual SRT task [36]; that is, learning on the SRT task is often minimally affected by cognitive load [36]. Concurrent tasks are argued to, however, the limit the availability of conscious knowledge [37], [38].

#### Performance on the post-training direct tests of sequence knowledge

Mean recognition confidence ratings for trained and untrained six-item sequences in experiments 2 and 3 are shown in Figure 2b. Remarkably, performance on the recognition test revealed that participants were able to recognise the masked trained second-order conditional sequence, even though the six-item trained and untrained sequences were identical when viewed binocularly, and differed only at the masked serial order (Figure 1d and Table 1). In particular, a repeated measures paired t-test revealed significant differences in mean confidence ratings between trained and untrained sequences in experiment 2 (*t*_(9)_ = 2.66, *p*<0.05). This result was replicated in experiment 3 where the difference in mean confidence ratings between trained and untrained sequences was also significant (*t*_(9)_ = 2.46, *p*<0.05). Furthermore, a mixed-factorial ANOVA performed on the mean recognition confidence ratings across both experiments indicates that the ability to recognise the trained (vs. untrained) sequences (*F*_(1,18)_ = 14.15, *p* = 0.001) was not modulated by orienting participants to the mapping between the binocular percept and the four-position array (*F*_(1,18)_ <1).

Interestingly, sequence knowledge was below the subjective threshold on the sequence awareness questionnaire: participants exhibited little or no knowledge about the target sequence (experiment 2, M rating on the awareness questionnaire  = 1.9, S.E.M. = 0.41; experiment 3, M rating on the awareness questionnaire  = 1.6, S.E.M. = 0.32; for rating scale, see Methods). Dissociations between subjective (e.g., a post-training awareness questionnaire) and objective (e.g., recognition test, free-generation task) measures of learning and awareness are often reported in other studies [10], and are argued to reveal gradations in the state of awareness associated with acquired knowledge or differences in the sensitivity of tests to acquired knowledge [39], [40]. Neither of these interpretations, however, is at variance with the conclusion that the ability to recognise trained, but masked, sequences provides evidence of newly acquired sequence-specific knowledge.

### Experiment 3: Effect of learning and test on the ability to consciously perceive the masked target locations

In agreement with the results from the LAT administered in experiment 1, the availability of target location information on the LAT administered in experiment 3 was at chance prior to training on the stereoscopic SL task (‘left’ targets: M = 0.46, S.E.M. = 0.05, *t*_(9)_ = −0.88, *p* = 0.40; ‘right’ targets: M = 0.50, S.E.M. = 0.05, *t*_(9)_ = 0.08, p = 0.94) and remained at chance when re-tested after the recognition test (‘left’ targets: M = 0.59, S.E.M. = 0.09, *t*_(9)_ = 1.08, p = 0.31; ‘right’ targets: M = 0.39, S.E.M. = 0.08, *t*_(9)_ = −1.40, *p* = 0.20). Furthermore, performance on the pre- and post-training LATs did not differ significantly (*t*_(9)_ = −1.39, p = 0.20 and *t*_(9)_ = 1.74, p = 0.12, for ‘left’ and ‘right’ targets, respectively). Similar findings were obtained after performing a signal detection analysis based on d' scores (see Table 3). Results from the LAT in experiment 1 and post-test LAT administered in experiment 3 thus reveal that participants were unable to identify the locations of masked targets even after extensive exposure to the second-order conditional sequence and an intentional orientation to the mapping between the four-position array and binocular percept. Importantly, even if the results had revealed an emerging, and reliable, ability to discriminate between any single pair of masked locations, it would only provide a source of noise because learning the masked second-order conditional sequence requires continuous interocular integration between each eye-of-origin and 9–12 blocks of (100) trials to reach asymptote.

**Table 3:** Mean of P(H), P(FA) and d' scores in the LAT test administered in Experiment 3.

Location Awareness Test: Pre-training/Post-recognition test	P(H)	P(FA)	d'	*t*_(9)_	*p*
Pre-training: ‘Left’	0.62	0.7	−0.25	−0.83	0.43
Pre-training: ‘Right’	0.74	0.55	−0.01	0.95	0.37
Post-testing: ‘Left’	0.45	0.44	0.85	−0.02	0.98
Post-testing: ‘Right’	0.42	0.63	−1.20	−1.06	0.32

One-sample t-tests indicate that sensitivity scores did not differ from chance (d' = 0).

## Discussion

The current results demonstrate that higher-order sequential associations can be learned via simple observation under conditions where observers are unable to consciously perceive the sequence of target locations or perform orienting responses that are correlated with the structure of the target sequence. Knowledge was expressed in the ability of participants to recognise the trained second-order conditional sequence, even though the trained and untrained recognition sequences were identical when viewed binocularly, and differed only at the masked serial order of the stimulus sequence. Furthermore, the ability to learn the masked visuospatial sequence was unaffected by prior knowledge of the mapping between the four-location array and appearance of targets within the binocular fused figure-of-eight. Together, therefore, the results reveal a heretofore-unknown capacity of the human visual system to support the detection, learning, and recognition of higher-order visuospatial associations that are masked from conscious awareness, under conditions where the response locations did not spatially correspond with the locations of the target sequence.

The ability to learn the visuospatial sequence under conditions where there is no correlation between the structure of the target and response sequence has been interpreted as evidence pure perceptual-based learning. Unlike prior studies, pure perceptual-based learning on the stereoscopic SL task involved acquiring knowledge about the structure of the spatial sequence of stimuli via the unconscious integration of retinotopic-based (allocentric [41]) codes stemming from monocular eye-of-origin information because the target sequence was based on interocular and higher-order visuospatial associations that were unavailable to conscious awareness. Importantly, knowledge about the four-location target sequence is unlikely to have been based on learning about the effects of a spatially-contingent response preceding each target (response-to-stimulus learning) [42], the response locations [41], [43], [44], stimulus-response associations [45], [46], the sequence of effector movements [47], integrated spatial/stimulus-response based sequence information [48], or the integration and organisation of action-effect codes into an ordered plan of actions [49], [50] because the responses were directed to two spatial locations (i.e., for SOC1, L R R L L R L R L R R L) versus the four locations of the target sequence (i.e., for SOC1, 3 4 2 3 1 2 1 4 3 2 4 1). By contrast, learning on perceptual-manual/oculomotor SRT tasks can involve one or more of these sources of information, depending on the experimental protocol. Indeed, it is conceivable that participants on the stereoscopic SL task also learned the sequence of response locations during training (for SOC1, L R R L L R L R L R R L), particularly given that the learning of response locations can be independent of learning a sequence of visuospatial locations [16], [17], [48]. Furthermore, the results raise an interesting issue regarding the role of attention on the stereoscopic SL task on the grounds that prior demonstrations of pure perceptual-based learning are dependent on the orienting of visuospatial attention (and possibly saccades) to the target stimuli [16]. Given the coupling between the systems that support eye movements and covert shifts of visuospatial attention [51], [52], [53], [54], additional insight into the role of visuospatial attention on stereoscopic SL task might be gained from analyses of eye movements, and microsaccades in particular [55], [56], [57], during training to examine involuntary responses to the visible and masked targets. More broadly, the experimental protocol provides a novel basis for exploring the learning of a sequence of visuospatial locations separately from learning response locations [17], [48].

Our results go beyond previous studies that have investigated the effects of unconscious visual stimuli on perceptual, semantic and motor repetition priming and other generally short-lived priming effects [58], [59], [60], [61] in three key areas: (1) unconscious processing was sufficient to support the learning of a masked higher-order visuospatial sequence of targets, presumably via an obligatory and elementary mechanism that is sensitive to associations between items [35], [62], [63]; (2) higher-order sequential associations between masked visual stimuli can be learned via interocular integration [64]; and (3) the resultant knowledge can support recognition memory. Importantly, however, even though learning was sufficient to support recognition memory under conditions where the retrieval context reinstated the conditions of the learning environment, accurate responding did not necessarily involve explicit knowledge. Recent evidence has shown that experience-dependent enhancements of perceptual fluency can lead to accurate responding on recognition tests that are more closely allied to perceptual priming than explicit memory [65], [66]. Hence, future investigation will need to establish the extent to which the products of learning on the stereoscopic SL task can be titrated along an implicit-explicit axis. One way in which to address this issue would be to manipulate parameters hypothesised to reduce the propensity for conscious awareness; these include reducing the amount of training [67], the availability of selective attention [68], and/or conduct training on a probabilistic, rather deterministic, second-order conditional sequence—stochastic noise reduces the likelihood of a target sequence being consciously detected [69]. Sequence knowledge acquired under these conditions could be assessed using not only direct tests but also using a concurrent indirect test of learning based on latency (priming) [10], [70] or the pattern of eye movements (including an analysis of microsaccades) to the targets [71]. Indeed, evidence of enhanced response (motor) fluency (priming) to trained (vs. untrained) sequences in the absence of recognition would be consistent with implicit knowledge [70], [71], [72]. However, in the absence of a correlation between the structure of the response and the target sequence, a priming based measure is unlikely to detect sequence knowledge and, more fundamentally, the (motor) response fluency associated with trained sequences is unlikely to contribute to “old” recognition ratings [70], [73].

The results also pose a challenge to neural models of learning and retrieval. The medial temporal lobe and related cortical regions, as part of a cortico-cerebellar/cortico-striatal network of regions identified with manual and oculomotor sequence learning [7], [8], [28], [74], are likely to be involved in supporting learning on the stereoscopic SL task. Activation in these regions has been identified with supporting the learning of second-order conditional associations [8], [28], [75], independently of the state of awareness associated with the acquired knowledge [7]. Hippocampal activation, however, has been shown to diminish in an adaptive manner, as learning reduces the demands related to the binding of higher-order dependencies [7]. Furthermore, even though activation in neural areas implicated in spatial response selection [76], [77] and the formation of spatial cue-to-response associations [78] are unlikely to correlate with sequence-specific learning on the grounds that the responses are uncorrelated with the target sequence, it is possible that learning on the stereoscopic SL task may correlate with activation in subcortical regions such as superior colliculus and lateral geniculate nucleus due to their hypothesised role in the selection of unconscious targets (i.e., such as in blindsight) [79], [80].

### Conclusions

Previous studies of human learning have focussed on the ability to detect and exploit relations between sequential visual targets that appear in close spatiotemporal proximity. A fundamental limitation with such demonstrations, however, is that learning has been assessed under conditions where the target sequence was consciously available. We show that higher-order sequential associations masked from conscious awareness can be learned through the unconscious interocular integration of retinotopic-based codes stemming from monocular eye-of-origin information and in the absence of spatially-contingent responses. Our experimental protocol, therefore, opens up a new approach to exploring the neurocognitive mechanisms and the role of awareness in the learning of sequential associations, which is of relevance to understanding cognitive faculties and behaviours as diverse as language acquisition, music, object knowledge formation, and motor learning.
